# Assessment of Gender-Based Linguistic Differences in Physician Trainee Evaluations of Medical Faculty Using Automated Text Mining

**DOI:** 10.1001/jamanetworkopen.2019.3520

**Published:** 2019-05-10

**Authors:** Janae K. Heath, Gary E. Weissman, Caitlin B. Clancy, Haochang Shou, John T. Farrar, C. Jessica Dine

**Affiliations:** 1 Department of Medicine, Perelman School of Medicine, University of Pennsylvania, Philadelphia; 2 Center for Clinical Epidemiology and Biostatistics, Perelman School of Medicine, University of Pennsylvania, Philadelphia; 3 Palliative and Advanced Illness Research Center, Perelman School of Medicine, University of Pennsylvania, Philadelphia; 4 Leonard Davis Institute of Health Economics, Perelman School of Medicine, University of Pennsylvania, Philadelphia; 5 Center for Healthcare Improvement and Patient Safety, Perelman School of Medicine, University of Pennsylvania, Philadelphia; 6 Perelman School of Medicine, Department of Biostatistics and Epidemiology, University of Pennsylvania, Philadelphia

## Abstract

**Importance:**

Women are underrepresented at higher ranks in academic medicine. However, the factors contributing to this disparity have not been fully elucidated. Implicit bias and unconscious mental attitudes toward a person or group may be factors. Although academic medical centers use physician trainee evaluations of faculty to inform promotion decisions, little is known about gender bias in these evaluations. To date, no studies have examined narrative evaluations of medical faculty by physician trainees for differences based on gender.

**Objective:**

To characterize gender-associated linguistic differences in narrative evaluations of medical faculty written by physician trainees.

**Design, Setting, and Participants:**

This retrospective cohort study included all faculty teaching evaluations completed for the department of medicine faculty by medical students, residents, and fellows at a large academic center in Pennsylvania from July 1, 2015, through June 30, 2016. Data analysis was performed from June 1, 2018, through July 31, 2018.

**Main Outcomes and Measures:**

Word use in faculty evaluations was quantified using automated text mining by converting free-text comments into unique 1- and 2-word phrases. Mixed-effects logistic regression analysis was performed to assess associations of faculty gender with frequencies of specific words and phrases present in a physician trainee evaluation.

**Results:**

A total of 7326 unique evaluations were collected for 521 faculty (325 men [62.4%] and 196 women [37.6%]). The individual words *art* (odds ratio [OR], 7.78; 95% CI, 1.01-59.89), *trials* (OR, 4.43; 95% CI, 1.34-14.69), *master* (OR, 4.24; 95% CI, 1.69-10.63), and *humor* (OR, 2.32; 95% CI, 1.44-3.73) were significantly associated with evaluations of male faculty, whereas the words *empathetic* (OR, 4.34; 95% CI, 1.56-12.07), *delight* (OR, 4.26; 95% CI, 1.35-13.40), and *warm* (OR, 3.45; 95% CI, 1.83-6.49) were significantly associated with evaluations of female faculty. Two-word phrases associated with male faculty evaluations included *run rounds* (OR, 7.78; 95% CI, 1.01-59.84), *big picture* (OR, 7.15; 95% CI, 1.68-30.42), and *master clinician* (OR, 4.02; 95% CI, 1.21-13.36), whereas evaluations of female faculty were more likely to be associated with *model physician* (OR, 7.75; 95% CI, 1.70-35.39), *just right* (OR, 6.97; 95% CI, 1.51-32.30), and *attention (to) detail* (OR, 4.26; 95% CI, 1.36-13.40).

**Conclusions and Relevance:**

The data showed quantifiable linguistic differences between free-text comments about male and female faculty in physician trainee evaluations. Further evaluation of these differences, particularly in association with ongoing gender disparities in faculty promotion and retention, may be warranted.

## Key Points

**Question:**

Are there gender-based linguistic differences in narrative evaluations of medical faculty written by physician trainees?

**Findings:**

In this cohort study of 7326 narrative evaluations for 521 medical faculty members, the words *art*, *trials*, *master*, and *humor* were significantly associated with evaluations of male faculty, whereas the words *empathetic*, *delight*, and *warm* were significantly associated with evaluations of female faculty. Two-word phrase associations included *run rounds*, *big picture*, and *master clinician* in male faculty evaluations and *model physician* and *attention (to) detail* in female faculty evaluations.

**Meaning:**

The findings suggest that quantitative linguistic differences between free-text comments about male and female medical faculty may occur in physician trainee evaluations.

## Teaser

This cohort study examines whether the specific word and phrase frequency used in physician trainee narrative evaluations of medical faculty and qualified by automated text mining is associated with the gender of the faculty member evaluated.

## Introduction

Although women compose half of all US medical school graduates,^1^ recent evidence has shown ongoing sex disparities in academic job achievements and academic promotion.^2,3,4,5,6,7,8^ Specifically, women in medicine represent only 38% of academic faculty and 15% of senior leadership positions across the United States.^9^ This underrepresentation of women has been attributed in part to high rates of attrition at multiple points along the promotion pathway. Although there is robust evidence^2,3,4,5,6,7,8,9^ showing the presence of these sex differences, the factors leading to disparities in faculty retention and promotion have not been fully elucidated.

One potential influence may be implicit bias, namely unconscious mental attitudes toward a person or group.^10^ Evidence suggests that implicit biases pervade academia, influencing hiring processes,^11^ mentorship,^12^ publication,^4^ and funding opportunities.^13,14,15^ In previous studies,^16,17,18,19^ implicit gender bias also has been quantitatively and qualitatively shown in evaluation of nonmedical academic faculty. Similar biases have also been noted in medical education within narrative evaluations of physician trainees written by medical faculty.^20,21^ However, despite the critical role of evaluations from physician trainees in promotion decisions for medical faculty, the presence of gender bias within evaluations of medical faculty written by physician trainees has not been adequately assessed.

Thus far, studies^22,23^ assessing gender differences in faculty evaluations have focused exclusively on numeric scales evaluating teaching effectiveness, with the largest such study showing no significant differences in numeric rating by gender. However, both numeric ratings and written comments are used by academic medical centers for promotion purposes. No studies, to our knowledge, have examined gender differences in narrative evaluations of medical faculty by physician trainees, which could identify implicit biases not captured by numeric ratings.

The use of computer-based language analysis or natural language processing techniques may provide a quantitative method to identify subtle linguistic differences in narrative evaluation that may not be captured by qualitative coding and thematic analysis. The practice of n-gram analysis, which converts text into a sequence of words and phrases for further quantitative analysis,^24^ has been successfully used to explore linguistic differences between genders in a variety of other settings.^25,26,27,28^ We hypothesized that use of an n-gram analysis could help quantify gender-associated differences in the vocabulary used by physician trainees in narrative evaluations of medical faculty.

## Methods

### Setting and Participants

We performed a single-center retrospective cohort analysis of all evaluations of department of medicine faculty that were written by physician trainees at a large academic medical center from July 1, 2015, to June 30, 2016. Anonymous evaluations of faculty, completed by medical students, residents, and fellows, consisted of a cumulative numeric score rating global teaching effectiveness (5-point rating scale: 1, poor; 2, fair; 3, good; 4, very good; and 5, excellent), in addition to mandated free-text comments. These evaluations were submitted by physician trainees within an anonymous online evaluation platform. Other than the global teaching effectiveness score, there were no separate ratings of faculty. Physician trainees completed the evaluations using a web-based platform at the end of each clinical rotation (both inpatient and outpatient), usually representing 1 to 2 weeks of exposure to the attending physician. The institutional review board of the University of Pennsylvania, Philadelphia, approved this study and granted a waiver of consent for participation. A waiver of consent was obtained because the research was retrospective and posed no more than minimal risk to participants, and the research could not be performed without a waiver. Our reporting adheres to the Strengthening the Reporting of Observational Studies in Epidemiology (STROBE) reporting guideline for cohort studies.

### Data Collection and Processing

The data set included faculty sex, faculty division within the department of medicine, physician trainee sex, physician trainee level (medical student, resident, or fellow), numeric rating scores (the teaching effectiveness score), and free-text comments. To preserve anonymity among the physician trainees, unique physician trainee identifiers were not associated with any evaluations. A third party deidentified all evaluations before the analysis. Text from the narrative portion of the evaluation was anonymized by replacing all first and last names with a placeholder (eg, *first name* and *last name*). In addition, text was converted to all lower case letters, and special characters were normalized. All punctuation was removed in the data preprocessing step, and stop words (such as *a*, *the*, and *this*) were removed from the text.

### N-gram Text Analysis

To analyze the narrative data, we used n-gram text mining to convert preprocessed free-text comments into discrete words and phrases. N-grams are 1- and 2-word phrases found in the text. We counted the presence of each n-gram in each document and then created a matrix of binary indicators for unigrams (single words) and bigrams (2-word phrases) for analysis. Because bigrams create phrases based on adjacent words within text, each unigram (discrete word) was represented in all n-gram categories. For example, the phrase “the attending taught the team skills” would be changed to “attending taught team skills” during preprocessing and then broken into 4 unigrams (*attending*, *taught*, *team*, and *skills*) and 3 bigrams (*attending taught*, *taught team*, and *team skills*). Bigrams were included to provide useful local contextual features that unigrams were unable to capture. N-grams occurring fewer than 10 times in the entire corpus were excluded from analysis because the study was not sufficiently powered to detect differences for such rarely occurring terms. Words describing a specific service (such as *cardiology* or *nephrology*), location (*medical intensive care unit*), or procedure (*bronchoscopy*) were excluded from analysis, given the potential effect of gender imbalance among faculty within specific specialties.

### Statistical Analysis

Data analysis was performed from June 1, 2018, through July 31, 2018. We assessed the associations of faculty gender with word or phrase use in the evaluations. The exposure variables were the frequency of words or phrases, as mined by the n-gram analysis. The dependent variable was faculty gender. Given the sparsity of the data matrix after initial n-gram text mining, variable selection was conducted using elastic net regression.^29,30^ Elastic net regression is a regularized regression method that eliminates insignificant covariates, preserves correlated variables, and guards against overfitting. To select an appropriate value for the tuning parameter (λ value), we used 10-fold cross-validation and selected the value that minimized the mean cross-validated error and reduced potential overfitting within the model. In addition to the aforementioned variables selected by elastic net regression, several unigrams were preselected for univariate analysis based on previous literature describing gender associations, specifically *teacher*, *first name*, and *last name*.^27^

The frequencies of selected unigram and bigram words were then fitted into a multivariable logistic regression model to assess their associations with faculty gender (using odds ratios [ORs] and 95% CIs). To assess the association of each individual n-gram with gender, each unigram and bigram was used in distinct mixed-effects logistic regression models (clustered on individual faculty members to assess the influence of instructor characteristics). To ensure anonymity of physician trainees, additional demographics (age and race/ethnicity) were not available in the data set, and unique physician trainee identifiers could not be used in the mixed-effects model. A secondary logistic regression was performed to evaluate the association of physician trainee gender with word choice.

For all analyses, the statistical significance was determined using the Benjamini-Hochberg approach for multiple comparisons, using a false discovery rate of 0.10, given the exploratory purposes of our analysis.^31,32^ This method controls for the false discovery rate using the analysis 2-sided *P* value compared with the Benjamini-Hochberg critical value ([*i*/*m*])*Q*, where *i* is the rank of the *P* value, *m* is the total number of tests, and *Q* is the false discovery rate.

Overall word counts by comment were tabulated for all evaluations in the cohort, and median word counts were compared across faculty gender, physician trainee gender, and physician trainee level using the Wilcoxon rank sum test (α = .05).

To evaluate quantitative differences between ratings by faculty gender, summary statistics were calculated across all submitted evaluations within the cohort. We performed a descriptive analysis of the overall teaching effectiveness score by comparing mean numeric ratings across faculty gender, physician trainee gender, and physician trainee level using the Wilcoxon rank sum test. All statistical analyses and additional text analysis were completed using Stata, version 15.1 (StataCorp).

## Results

### Demographic Characteristics

A total of 7326 unique evaluations were collected during the 2015 through 2016 academic year for 521 faculty members in the Department of Medicine at the Perelman School of Medicine, University of Pennsylvania, Philadelphia, including 325 men (62.4%) and 196 women (37.6%). Complete data (including the sex of the faculty member and physician trainee) were available for 6840 (93.4%) evaluations. Individual faculty members had a mean (SD) of 14.1 (13.8) evaluations (range, 1.0-76.0) included in the cohort. Most of the evaluations (5732 [78.2%]) were submitted by physicians in residency or fellowship training, and the remainder were submitted by medical students during clerkship, elective, and subinternship rotations (Table). Male physician trainees completed 3724 of 6840 faculty evaluations (50.8%) with complete data.

**Table.** Demographic Information of Faculty Evaluation Cohort

Characteristic	No. (%)	
	Total (N = 521)	Evaluations (n = 6840)^a^
Faculty		
Male identified	325 (62.4)	4448 (61)
Female identified	196 (37.6)	2878 (39)
Physician trainees^b^		
Male identified	NA	3724 (54.4)
Female identified	NA	3116 (46)
Medical students	NA	1594 (22)
Residents or fellows	NA	5732 (78)
Division		
General medicine	111 (21.3)	1818 (26.6)
Cardiology	88 (16.9)	1335 (19.5)
Gastroenterology	51 (9.8)	558 (8.2)
Geriatrics	14 (2.7)	137 (2.0)
Hematology/oncology	76 (14.6)	642 (9.4)
Infectious diseases	47 (9.0)	485 (7.1)
Pulmonary, allergy, and critical care	62 (11.9)	1217 (17.8)
Rheumatology	25 (4.8)	264 (3.8)
Renal	36 (6.9)	332 (4.8)
Sleep medicine	11 (2.1)	52 (0.7)

Abbreviation: NA, not applicable.

^a^ Of the 7326 evaluations, 6840 had full data on both medical faculty and physician trainee gender.

^b^ No unique physician trainee identifiers were captured within the data set to preserve physician trainee anonymity of evaluation.

Faculty members (N = 521) represented all of the following divisions within the department of medicine: general medicine (n = 111; 21.3%); cardiology (n = 88; 16.9%); gastroenterology (n = 51; 9.8%); geriatrics (n = 14; 2.7%); hematology and oncology (n = 76; 14.6%); infectious diseases (n = 47; 9.0%); pulmonary, allergy, and critical care (n = 62; 11.9%); rheumatology (n = 25; 4.8%); renal (n = 36; 6.9%); and sleep medicine (n = 11; 2.1%).

### N-gram Text Analysis

After removal of stop words, n-gram text analysis was used to extract text features, creating a matrix of binary indicators for 1610 unigrams and 1698 bigrams. Variable selection with elastic net regression identified a subset of 151 unigrams and 165 bigrams for further analysis. The complete list of selected variables is included in eTable 1 and eTable 2 in the Supplement.

**Figure 1.**
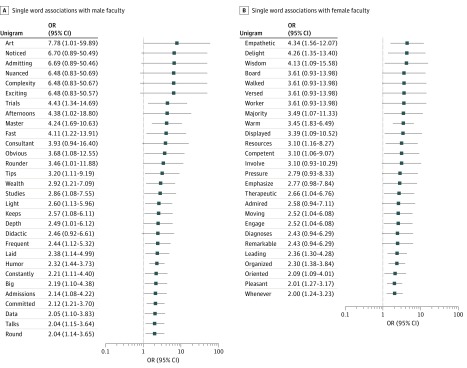
Significant Single-Word (Unigram) Associations by Faculty Gender OR indicates odds ratio.

Our findings showed that written evaluations including *art* (OR, 7.78; 95% CI, 1.01-59.89), *trials* (OR, 4.43; 95% CI, 1.34-14.69), *master* (OR, 4.24; 95% CI, 1.69-10.63), and *humor* (OR, 2.32; 95% CI, 1.44-3.73) were associated with evaluations of male faculty, whereas words including *empathetic* (OR, 4.34; 95% CI, 1.56-12.07), *delight* (OR, 4.26; 95% CI, 1.35-13.40), and *warm* (OR, 3.45; 95% CI, 1.83-6.49) were associated with evaluations of female faculty. Figure 1 highlights all significant single word associations with faculty gender (based on Benjamini-Hochberg threshold). The full list of unigrams that were associated with gender are listed in eTable 1 in the Supplement. Analysis of previously identified words associated with gender, including *teacher*, *first name*, and *last name,* did not reveal significant associations with faculty gender.

**Figure 2.**
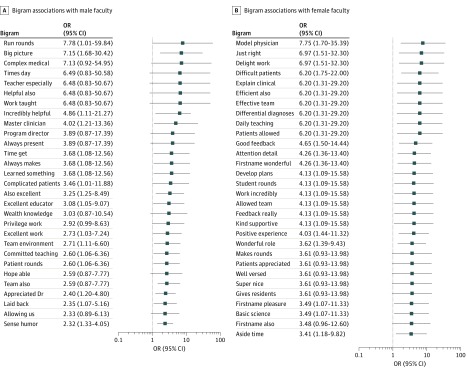
Significant 2-Word (Bigram) Associations by Faculty Gender OR indicates odds ratio.

The 2-word phrases that were associated with male faculty evaluations included *run rounds* (OR, 7.78; 95% CI, 1.01-59.84), *big picture* (OR, 7.15; 95% CI, 1.68-30.42), and *master clinician* (OR, 4.02; 95% CI, 1.21-13.36). Female faculty evaluations tended to contain more comments like *model physician* (OR, 7.75; 95% CI, 1.70-35.39), *just right* (OR, 6.97; 95% CI, 1.51-32.30), and *attention (to) detail* (OR, 4.26; 95% CI, 1.36-13.40). All 2-word phrases significantly associated with gender are displayed in Figure 2. The full bigram associations are included in eTable 2 in the Supplement. A secondary analysis did not reveal significant differences in word choice based on the gender of the physician trainee after adjustment for multiple comparisons (eTable 3 in the Supplement)

### Descriptive Analysis of Narrative Comments and Numeric Rating (Teaching Effectiveness Score)

Narrative evaluations had a median word count of 32 (IQR, 12-60 words). The median word count was significantly higher for evaluations of female faculty (33 words; IQR, 14-62 words) compared with evaluations of male faculty (30 words; IQR, 12-58 words) (*P* < .001). Neither resident gender nor training level was significantly associated with narrative evaluation word count.

There was no significant difference in the mean numeric ratings of overall teaching effectiveness between female and male faculty (mean difference, 0.02; 95% CI, –0.05 to 0.03). There was also no significant difference between mean numeric ratings assigned by female and male residents or fellows (0.02; 95% CI, –0.05 to 0.03). Compared with residents, medical students assigned significantly higher ratings to the faculty (0.22; 95% CI, 0.17-0.26; *P* < .001).

## Discussion

Our data showed quantitative linguistic differences in free-text comments based on faculty gender that persisted after adjustment for evaluator gender and level of training. These word choice differences, while not innately negative, reflected notable contrasts in the ways male and female faculty were perceived and evaluated. These differences are particularly relevant if job qualifications are defined using stereotypically male characteristics, which may preclude women from these opportunities if evaluations use different descriptors. To our knowledge, this was the first study to focus on narrative evaluation of faculty within the medical field. Because narrative evaluations are frequently used to guide decisions about reappointment, promotion, salary increases, and consideration for leadership positions, bias in these evaluations may have far-reaching consequences for the career trajectories of medical faculty.

The word associations identified in our cohort affirmed linguistic trends by gender previously reported in nonmedical settings,^11,12,16,17,18,19,33,34,35^ specifically the use of ability terms (such as *master* and *complexity*) associated with men and emotive terms (such as *empathetic*, *delight*, and *warm*) associated with women. This mirrors previous qualitative work^21,33,34,35^ highlighting gender-based differences in patterns of word choice used in letters of recommendation and performance evaluations that identified differential use of grindstone traits (eg, hardworking), ability traits, and standout adjectives (eg, best). Our results were also consistent with previous findings in other educational settings where trainees (irrespective of age and generational characteristics) displayed biases in ratings of teachers.^16,19,20^ The linguistic differences noted in our study suggest the need for further evaluation of potential biases in narrative evaluations of faculty.

Despite the linguistic differences, we did not identify differences in numeric rating of teaching effectiveness by faculty gender. We suspect that numeric rating, given the significant positive skew and limited spread of the ratings, may be insensitive to the magnitude and/or types of differences associated with gender bias in this setting. However, this limited range of ratings may suggest greater importance to the written comments, and differential word choice may reveal subtle imbalances not appreciated in ratings data. Although the numeric ratings were equal, there were differences in the physician trainee descriptors used in evaluation of faculty, and there may be differential valuation of the mentioned characteristics by academic medical centers. Specifically, recent evidence^36^ highlights that the presence of humor is significantly associated with performance evaluation and assessment of leadership capability. Further study on how the different descriptors used in faculty evaluation are associated with performance appraisal and promotion in academic medical centers is warranted.

Although previous qualitative research has shown the presence of bias in narrative evaluation of physician trainees, this study offers novel insights into the potential for gender bias throughout the spectrum of narrative evaluations in medicine. In addition, the use of natural language processing techniques enabled a robust analysis of a large number of evaluations, exposing word choice differences that may not have been perceived by using qualitative techniques alone. Further study is warranted to explore the effect of gender-based linguistic differences in narrative evaluations on decisions about reappointment, promotion, and salary increases. Clarifying the ultimate effect of these differences on career trajectories of women in medicine may help identify interventions (such as implicit bias training for physician trainees and/or designing faculty evaluation forms to encourage the use of standard descriptors for both genders) to prevent or mitigate implicit bias.

### Strengths and Limitations

The strengths of our study included the size and composition of our faculty cohort, representing all clinical departments within the department of medicine, as well as the inclusion of physician trainee raters spanning from clinical undergraduate medical education (medical school) to graduate medical education (residency and fellowship). In addition, the use of n-gram analysis allowed for objective quantification of word use within the sample. Although n-gram analysis may be limited for capturing important phrases or context, other natural language processing methods, such as embedding vectors or recurrent neural network models, may be more appropriate and an important area of future work. Further qualitative research may be warranted to examine the context of the word choice differences identified in this study.

A major limitation of the current study is generalizability, since our data set included faculty in the department of medicine within a single large academic institution. However, because the cohort consisted of narrative evaluations written by physician trainees from various geographic backgrounds and previous training institutions, we believe that the results are likely to be representative of academic faculty evaluations at other institutions. Future work may be warranted to determine whether similar associations exist in specialties other than internal medicine and its subspecialties.

Although we were unable to ascertain objective differences in attributes of faculty based on gender, our study highlights that there are certainly differences in which attributes are perceived by physician trainee evaluators. It is possible that existing biases about the inherent value of these characteristics may play a role in interpretation and valuation of these comments by promotion committees. Also, we were not able to assess the effect of underrepresented minority status, given the small numbers of faculty and physician trainees within our institution with this status as well as the observational nature of this study. Although our n-gram analysis identified differences in unigram and bigram descriptors applied to male vs female faculty members' performance, it did not fully capture contextual differences in comments, which would require additional qualitative study and/or application of additional computational techniques.

## Conclusions

This study identified quantitative linguistic differences in physician trainee evaluations of faculty on the basis of gender. Stereotypical gender phrases were identified in physician trainee evaluations of medical faculty (such as ability and cognitive terms in male faculty evaluations and emotive terms in female faculty evaluations). There were no significant differences in numeric ratings between female and male faculty members, which may add greater importance to the language used in narrative comments. Overall, these findings suggest the presence of gender differences in narrative evaluations of faculty written by physician trainees. Further research may be warranted to explore the context of the specific words and phrases identified and to understand the downstream effect of such evaluation differences on the career trajectories of women in medicine.

## Notes

Supplement. **eTable 1.** Unigram Associations With Female Faculty Sex (Following Variable Selection With Elastic Net Regression) **eTable 2.** Bigram Associations With Female Faculty Sex (Following Variable Selection With Elastic Net Regression) **eTable 3.** Secondary Analysis of Unigram Association With Trainee Female Sex
