# Supplementary material for: Assessment of Gender-Based Linguistic Differences in Physician Trainee Evaluations of Medical Faculty Using Automated Text Mining
Source: JAMA Netw Open. 2019 May 10;2(5):e193520. doi: 10.1001/jamanetworkopen.2019.3520 (PMC6512459; doi:10.1001/jamanetworkopen.2019.3520)
Supplement: Supplement. — eTable 1. Unigram Associations With Female Faculty Sex (Following Variable Selection With Elastic Net Regression) eTable 2. Bigram Associations With Female Faculty Sex (Following Variable Selection With Elastic Net Regression) eTable 3. Secondary Analysis of Unigram Association With Trainee Female Sex [file jamanetwopen-2-e193520-s001.pdf]

Supplementary Online Content

Heath JK, Weissman GE, Clancy CB, Shou H, Farrar JT, Dine CJ. Assessment of gender-based linguistic differences in physician trainee evaluations of medical faculty using automated text mining. *JAMA Netw Open*. 2019;2(5):e193520. doi:10.1001/jamanetworkopen.2019.3520

eTable 1. Unigram Associations With Female Faculty Sex (Following Variable Selection With Elastic Net Regression)

eTable 2. Bigram Associations With Female Faculty Sex (Following Variable Selection With Elastic Net Regression)

eTable 3. Secondary Analysis of Unigram Association With Trainee Female Sex

This supplementary material has been provided by the authors to give readers additional information about their work.

© 2019 Heath JK et al. *JAMA Network Open.*

eTable 1. Unigram Associations with Female Faculty Sex (Following Variable Selection with Elastic Net Regression)

Unigram	OR	Confidence Interval		P-value	Hochberg Significance Threshold
Art	0.13	0.02	0.99	0.05	0.05
Admitting	0.15	0.02	1.13	0.06	0.06
Complexity	0.15	0.02	1.21	0.07	0.07
Exciting	0.15	0.02	1.21	0.07	0.07
Noticed	0.15	0.02	1.13	0.07	0.06
Nuanced	0.15	0.02	1.21	0.07	0.07
Tips	0.21	0.11	0.90	0.03	0.03
Afternoons	0.23	0.05	0.98	0.05	0.05
Trials	0.23	0.07	0.75	0.02	0.02
Fast	0.24	0.07	0.82	0.02	0.02
Master	0.24	0.09	0.59	0.002	0.003
Consultants	0.25	0.06	1.06	0.06	0.05
Easier	0.25	0.01	11.23	0.48	0.09
Obvious	0.27	0.08	0.93	0.04	0.04
Nurses	0.27	0.001	42.74	0.61	0.10
Hold	0.28	0.06	1.27	0.10	0.08
Journal	0.28	0.06	1.27	0.10	0.08
Rounder	0.29	0.08	0.99	0.05	0.05
Behavior	0.31	0.07	1.41	0.13	0.09
Procedure	0.31	0.07	1.41	0.13	0.09
Shadow	0.31	0.07	1.41	0.13	0.09
Wealth	0.34	0.14	0.83	0.02	0.02
Studies	0.35	0.13	0.93	0.03	0.03
Possibly	0.35	0.08	1.52	0.16	0.09
Stuff	0.36	0.11	1.22	0.10	0.08
Light	0.38	0.17	0.88	0.02	0.02
Keeps	0.39	0.16	0.92	0.03	0.03
Humble	0.39	0.13	1.15	0.09	0.07
Depth	0.40	0.16	0.99	0.05	0.05
Frequent	0.41	0.19	0.90	0.03	0.03
Didactic	0.41	0.15	1.09	0.07	0.06
Placed	0.41	0.14	1.24	0.12	0.09
Laid	0.42	0.20	0.88	0.02	0.02
Humor	0.43	0.27	0.69	0.001	0.001

© 2019 Heath JK et al. *JAMA Network Open.*

Unigram	OR	Confidence Interval		P-value	Hochberg Significance Threshold
Hearing	0.43	0.16	1.15	0.09	0.07
Big	0.45	0.23	0.91	0.03	0.03
Constantly	0.45	0.23	0.90	0.02	0.02
Engaged	0.45	0.03	6.52	0.56	0.10
Pointed	0.46	0.13	1.68	0.24	0.09
Admissions	0.47	0.24	0.93	0.03	0.03
Committed	0.47	0.27	0.82	0.008	0.01
Talks	0.48	0.27	0.87	0.02	0.02
Data	0.49	0.26	0.91	0.02	0.02
Round	0.49	0.27	0.88	0.02	0.02
Ideal	0.51	0.20	1.17	0.11	0.08
Workload	0.51	0.19	1.42	0.20	0.09
Research	0.53	0.29	0.94	0.03	0.03
Fostered	0.53	0.25	1.11	0.09	0.07
Personality	0.53	0.25	1.14	0.11	0.08
Emphasis	0.55	0.29	1.04	0.07	0.06
Post	0.56	0.31	1.01	0.05	0.05
Someone	0.57	0.30	1.06	0.08	0.07
Demeanor	0.58	0.32	1.05	0.07	0.06
Lectures	0.58	0.37	0.92	0.02	0.02
Multiple	0.59	0.36	0.96	0.03	0.04
Otherwise	0.60	0.34	1.07	0.09	0.08
Privilege	0.6	0.35	1.04	0.07	0.06
Knowledgeable	0.61	0.38	0.99	0.05	0.05
Hands	0.62	0.38	1.01	0.06	0.05
Procedures	0.62	0.39	1.00	0.05	0.05
Seemed	0.62	0.09	4.06	0.62	0.10
Aspects	0.63	0.36	1.07	0.09	0.08
Real	0.63	0.39	1.00	0.05	0.05
Long	0.67	0.48	0.94	0.02	0.03
Based	0.70	0.52	0.94	0.02	0.03
Found	0.74	0.52	1.05	0.09	0.08
Evidence	0.75	0.57	0.99	0.04	0.04
Able	0.76	0.62	0.94	0.01	0.01
Incorporate	0.76	0.45	1.29	0.31	0.09
Members	0.76	0.56	1.02	0.07	0.06

© 2019 Heath JK et al. *JAMA Network Open.*

Unigram	OR	Confidence Interval		P-value	Hochberg Significance Threshold
Fellow	0.80	0.64	1.01	0.06	0.05
Knowledge	0.81	0.68	.97	0.03	0.04
Teacher	0.83	0.75	0.93	0.001	0.002
Get	0.84	0.69	1.04	0.11	0.08
Provided	0.84	0.66	1.08	0.18	0.09
Fellows	0.87	0.71	1.06	0.17	0.09
Patients	1.11	0.77	1.61	0.57	0.10
Time	1.12	1.04	1.21	0.005	0.008
Working	1.13	1.03	1.25	0.01	0.01
Service	1.15	1.01	1.31	0.03	0.04
Work	1.16	0.69	1.97	0.57	0.10
One	1.18	1.04	1.34	0.01	0.01
Pleasure	1.19	1.02	1.39	0.03	0.04
Plan	1.19	0.97	1.46	0.10	0.08
Spend	1.20	0.78	1.83	0.41	0.09
Amazing	1.21	0.99	1.47	0.06	0.06
Management	1.22	0.95	1.50	0.06	0.06
Wonderful	1.22	1.04	1.44	0.02	0.03
Plans	1.23	0.98	1.56	0.08	0.07
Hope	1.24	1.02	1.51	0.03	0.04
Interns	1.25	1.02	1.53	0.03	0.04
Make	1.28	1.08	1.52	0.004	0.007
Enthusiastic	1.30	0.94	1.78	0.11	0.08
Supportive	1.30	1.08	1.54	0.004	0.008
Approachable	1.33	1.08	1.63	0.008	0.01
Model	1.33	1.09	1.61	0.005	0.009
Fantastic	1.34	1.10	1.62	0.003	0.005
Comfortable	1.35	1.02	1.79	0.04	0.04
Busy	1.37	1.11	1.70	0.003	0.005
Friendly	1.40	1.02	1.92	0.04	0.04
Rapport	1.40	0.92	2.14	0.12	0.09
Still	1.41	1.05	1.89	0.02	0.03
Career	1.43	0.95	2.16	0.08	0.07
Favorite	1.44	1.02	2.02	0.04	0.04
Nice	1.44	1.07	1.94	0.02	0.03
Allowed	1.47	1.18	1.84	0.001	0.002

© 2019 Heath JK et al. *JAMA Network Open.*

Unigram	OR	Confidence Interval		P-value	Hochberg Significance Threshold
Failure	1.49	0.97	2.23	0.07	0.06
Expectations	1.49	1.09	2.03	0.01	0.01
Person	1.50	1.04	2.15	0.02	0.03
Although	1.53	0.96	2.46	0.08	0.07
Wish	1.53	1.06	2.04	0.02	0.03
Compassionate	1.60	1.07	2.36	0.02	0.03
Lead	1.62	1.12	2.32	0.01	0.02
Month	1.62	0.98	2.69	0.06	0.06
Preceptor	1.63	1.18	2.26	0.003	0.006
Inspiring	1.64	0.98	2.76	0.06	0.06
Question	1.65	1.04	2.64	0.03	0.04
Encouraged	1.67	1.20	2.31	0.002	0.004
Attitude	1.69	1.12	2.56	0.01	0.02
Follow	1.73	1.03	2.91	0.04	0.04
Want	1.73	1.06	2.82	0.02	0.03
Structured	1.73	0.82	3.67	0.15	0.09
Strong	1.74	1.18	2.58	0.005	0.009
Short	1.75	1.14	2.67	0.01	0.02
Detail	1.82	1.14	2.91	0.01	0.02
Mind	1.84	0.96	3.59	0.07	0.07
Glad	1.89	0.98	3.68	0.06	0.06
Benefit	1.92	0.96	3.80	0.06	0.06
Independent	1.93	1.14	3.27	0.01	0.02
Meet	1.97	0.89	4.35	0.09	0.08
Prepared	1.97	0.89	4.35	0.09	0.08
Seeing	1.99	1.18	3.36	0.01	0.02
Smoothly	1.99	0.99	4.01	0.05	0.05
Whenever	2.00	1.24	3.23	0.005	0.01
Pleasant	2.01	1.27	3.17	0.003	0.006
SAR	2.03	0.88	4.70	0.10	0.08
Oriented	2.10	1.09	4.01	0.03	0.04
Suggest	2.17	0.69	6.82	0.19	0.09
Believe	2.21	0.24	3.43	0.49	0.10
Positive	2.21	1.42	33.33	0.57	0.10
Proactive	2.21	0.84	5.82	0.11	0.08
Organized	2.30	1.38	3.84	0.001	0.003

© 2019 Heath JK et al. *JAMA Network Open.*

Unigram	OR	Confidence Interval		P-value	Hochberg Significance Threshold
Previous	2.32	0.83	6.53	0.11	0.08
Leading	2.36	1.3	4.28	0.005	0.01
Diagnoses	2.43	0.94	6.29	0.07	0.07
Remarkable	2.43	0.94	6.29	0.07	0.07
Initially	2.48	0.81	7.58	0.11	0.08
Styles	2.48	0.81	7.58	0.11	0.09
Engage	2.52	1.04	6.08	0.04	0.04
Moving	2.52	1.04	6.08	0.04	0.05
Admired	2.58	0.94	7.11	0.07	0.07
Therapeutic	2.66	1.04	6.76	0.04	0.05
Emphasize	2.77	0.98	7.84	0.05	0.05
Pressure	2.79	0.93	8.33	0.07	0.07
Documentation	3.10	0.93	10.29	0.07	0.07
Involve	3.10	0.93	10.29	0.07	0.07
Competent	3.10	1.06	9.07	0.04	0.05
Resources	3.10	1.16	8.27	0.02	0.03
Displayed	3.39	1.09	10.52	0.03	0.04
Warm	3.45	1.83	6.49	<0.0001	0.001
Via	3.49	0.14	124.65	0.42	0.09
Majority	3.49	1.07	11.33	0.04	0.05
Board	3.61	0.93	13.98	0.06	0.06
Versed	3.61	0.93	13.98	0.06	0.06
Walked	3.61	0.93	13.98	0.06	0.06
Worker	3.61	0.93	13.98	0.06	0.06
Wisdom	4.13	1.09	15.58	0.03	0.04
Delight	4.26	1.35	13.40	0.01	0.02
Empathetic	4.34	1.56	12.07	0.005	0.01
Text	4.54	0.06	336.73	0.49	0.10
Couple	9.08	0.01	7189.03	0.52	0.10

© 2019 Heath JK et al. *JAMA Network Open.*

eTable 2. Bigram Associations with Female Faculty Sex (Following Variable Selection with Elastic Net Regression)

Bigram	OR	Confidence Interval		P-value	Hochberg Significance Threshold
Run Rounds	0.13	0.02	0.99	0.05	0.06
Much Dr	0.14	0.30	0.99	0.01	0.06
Big Picture	0.14	0.03	0.60	0.01	0.01
Complex Medical	0.14	0.02	1.08	0.06	0.06
Helpful Also	0.15	0.02	1.21	0.08	0.08
Teacher Especially	0.15	0.02	1.21	0.08	0.08
Times Day	0.15	0.02	1.21	0.08	0.08
Work Taught	0.16	0.02	1.21	0.08	0.08
Always Finds	0.17	0.02	1.35	0.09	0.09
Clinical Trials	0.17	0.02	1.36	0.09	0.09
Well Read	0.17	0.02	1.35	0.09	0.09
Last-name Team	0.17	0.02	1.35	0.09	0.09
Often Times	0.17	0.02	1.35	0.09	0.09
Incredibly Helpful	0.21	0.05	0.90	0.04	0.04
Last-name Lot	0.21	0.05	0.90	0.04	0.04
Wealth Knowledge	0.22	0.09	1.15	0.08	0.09
Master Clinician	0.24	0.07	0.82	0.02	0.03
Program Director	0.26	0.06	1.15	0.08	0.08
Always Present	0.26	0.06	1.15	0.08	0.08
Time Get	0.27	0.08	0.92	0.04	0.05
Always Makes	0.27	0.08	0.93	0.04	0.04
Learned Something	0.27	0.08	0.93	0.04	0.05
Patients Think	0.28	0.06	1.27	0.10	0.09
Supportive Learning	0.28	0.06	1.27	0.10	0.09
Complicated Patients	0.29	0.08	0.99	0.05	0.06
Also Excellent	0.31	0.12	0.81	0.02	0.02
Communicating Patients	0.31	0.07	1.41	0.13	0.10
Allowing Us	0.32	0.11	0.95	0.04	0.05
Excellent Educator	0.32	0.11	0.96	0.04	0.05
Privilege Work	0.34	0.12	1.01	0.05	0.06
Team Environment	0.36	0.15	0.90	0.03	0.03
Excellent Work	0.37	0.14	0.97	0.04	0.05
Committed Teaching	0.39	0.16	0.94	0.04	0.04
Patients Rounds	0.39	0.16	0.94	0.04	0.04

© 2019 Heath JK et al. *JAMA Network Open.*

Bigram	OR	Confidence Interval		P-value	Hochberg Significance Threshold
Team Also	0.39	0.13	1.15	0.09	0.09
Hope Able	0.39	0.13	1.15	0.09	0.09
Able Learn	0.41	0.14	1.21	0.12	0.10
Patient Family	0.41	0.14	1.24	0.12	0.10
Appreciated Dr	0.42	0.21	0.84	0.01	0.02
Laid Back	0.42	0.19	0.93	0.03	0.03
Sense Humor	0.43	0.25	0.75	0.003	0.005
Teaching Always	0.45	0.21	0.94	0.03	0.04
Enjoys Teaching	0.46	0.19	1.15	0.10	0.09
Great Clinical	0.47	0.21	1.05	0.07	0.07
Asking Questions	0.51	0.25	1.05	0.07	0.08
Goals Care	0.54	0.29	1.02	0.06	0.06
Spent Time	0.59	0.21	1.67	0.32	0.10
Team Members	0.60	0.34	1.04	0.07	0.08
Evidence Based	0.62	0.42	0.93	0.02	0.02
Teaching Sessions	0.66	0.41	1.06	0.09	0.09
Excellent Teacher	0.68	0.54	0.85	0.001	0.001
Two Weeks	0.71	0.45	1.14	0.16	0.10
Great Teacher	0.83	0.67	1.01	0.07	0.08
Working Dr	1.14	0.98	1.32	0.08	0.08
Pleasure Work	1.23	1.01	1.50	0.04	0.04
Role Model	1.40	1.13	1.73	0.002	0.004
Time Teaching	1.40	1.02	1.96	0.04	0.04
Last-name Wonderful	1.44	1.01	2.05	0.05	0.05
Look Forward	1.47	1.11	1.95	0.01	0.01
Care Patients	1.52	1.05	2.22	0.03	0.03
Fantastic Teacher	1.55	0.99	2.43	0.05	0.06
Always Pleasure	1.55	0.91	2.65	0.11	0.10
Last-name Fantastic	1.58	1.14	2.19	0.01	0.01
Opportunity Work	1.59	1.08	2.33	0.02	0.02
One Favorites	1.63	1.07	2.46	0.02	0.03
Wonderful Attending	1.72	1.02	2.91	0.04	0.05
Hope Work	1.76	1.22	2.52	0.002	0.003
Clinical Care	1.84	0.95	3.59	0.07	0.08
Take Time	1.89	1.02	3.54	0.05	0.05
Teacher Clinician	1.89	1.02	3.50	0.05	0.06

© 2019 Heath JK et al. *JAMA Network Open.*

Bigram	OR	Confidence Interval		P-value	Hochberg Significance Threshold
Pleasant Work	1.91	0.92	3.97	0.08	0.09
One Day	1.92	1.01	3.63	0.05	0.06
Patients Care	1.97	0.89	4.34	0.09	0.09
Time Day	2.01	1.06	3.90	0.03	0.04
Senior Resident	2.10	1.19	3.93	0.02	0.03
Rapport Patients	2.12	1.22	3.68	0.01	0.01
Clear Expectations	2.17	0.96	4.89	0.06	0.06
Short Time	2.23	0.96	5.24	0.06	0.07
Patients Service	2.25	1.05	4.86	0.04	0.05
Last-name Absolutely	2.32	0.95	5.69	0.07	0.07
Opportunity Learn	2.32	0.94	5.69	0.07	0.07
Differential Diagnosis	2.32	0.83	6.53	0.11	0.10
Last-name Enthusiastic	2.33	0.95	5.69	0.07	0.07
Clinical Educator	2.41	1.04	5.57	0.04	0.05
Always Open	2.43	0.94	6.28	0.07	0.07
Supportive Fellows	2.43	0.94	6.28	0.07	0.07
Way Teach	2.43	0.94	6.28	0.07	0.08
Work Really	2.51	1.04	6.08	0.04	0.05
Enthusiastic Teaching	2.53	1.20	5.38	0.02	0.02
Care Patient	2.58	0.94	7.11	0.07	0.08
New Patient	2.58	0.94	7.11	0.07	0.08
True Pleasure	2.60	0.94	7.11	0.07	0.08
Teacher Attending	2.70	0.93	8.32	0.07	0.07
Dedicated Patients	2.71	1.14	6.48	0.03	0.03
Detail Oriented	2.71	1.14	6.48	0.03	0.03
Made Great	2.71	0.79	9.26	0.11	0.10
Us Lot	2.74	1.39	5.43	0.004	0.01
Lead Team	2.76	1.22	6.24	0.02	0.02
Service Busy	2.84	1.05	7.69	0.04	0.05
Ownership Patients	2.89	1.15	7.22	0.02	0.03
Positive Attitude	3.09	1.16	8.26	0.02	0.03
Commitment Teaching	3.09	0.93	10.29	0.07	0.07
Excellent Care	3.09	0.93	10.30	0.07	0.07
Involved Patient	3.10	1.33	7.25	0.01	0.01
Students Interns	3.10	1.25	7.69	0.02	0.02
Went Beyond	3.10	1.06	9.09	0.04	0.05

© 2019 Heath JK et al. *JAMA Network Open.*

Bigram	OR	Confidence Interval		P-value	Hochberg Significance Threshold
Always Feel	3.10	0.93	10.30	0.07	0.07
First Time	3.35	1.28	8.84	0.01	0.02
Relationship Patients	3.40	1.18	9.82	0.02	0.03
Aside Time	3.41	1.18	9.82	0.02	0.03
Basic Science	3.49	1.07	11.33	0.04	0.05
First-name Pleasure	3.49	1.07	11.33	0.04	0.05
Wonderful Role	3.61	1.39	9.43	0.01	0.01
First-name Also	3.61	0.93	13.98	0.06	0.06
Gives Residents	3.61	0.93	13.98	0.06	0.06
Makes Rounds	3.61	0.93	13.98	0.06	0.06
Patients Appreciated	3.61	0.93	13.98	0.06	0.07
Super Nice	3.61	0.93	13.98	0.06	0.07
Well Versed	3.61	0.93	13.98	0.06	0.07
Positive Experience	4.03	1.44	11.32	0.01	0.01
Allowed Team	4.13	1.09	15.58	0.04	0.04
Develop Plans	4.13	1.09	15.58	0.04	0.04
Feedback Really	4.13	1.09	15.58	0.04	0.04
Kind Supportive	4.13	1.09	15.58	0.04	0.04
Students Rounds	4.13	1.09	15.58	0.04	0.04
Work Incredibly	4.13	1.09	15.58	0.04	0.04
Attention Detail	4.26	1.36	13.40	0.01	0.02
First-name Wonderful	4.26	1.36	13.40	0.01	0.02
Good Feedback	4.65	1.50	14.44	0.01	0.01
Daily Teaching	6.20	1.31	29.20	0.02	0.02
Differential Diagnoses	6.20	1.31	29.20	0.02	0.02
Efficient Also	6.20	1.31	29.20	0.02	0.03
Explain Clinical	6.20	1.31	29.20	0.02	0.03
Difficult Patients	6.20	1.75	22.03	0.01	0.01
Effective Team	6.20	1.31	29.20	0.02	0.02
Patients Allowed	6.20	1.31	29.20	0.02	0.03
Delight Work	6.97	1.51	32.30	0.01	0.02
Just Right	6.97	1.51	32.30	0.01	0.02
Model Physician	7.75	1.70	35.39	0.01	0.01

© 2019 Heath JK et al. *JAMA Network Open.*

eTable 3. Secondary Analysis of Unigram Association with Trainee Female Sex

Unigram	OR	Confidence Interval		P-value
Art	0.75	0.25	2.28	0.61
Admitting	2.38	0.72	7.92	0.18
Complexity	1.99	0.48	8.35	0.35
Exciting	1.20	0.35	4.16	0.77
Noticed	0.88	0.20	2.33	0.54
Nuanced	0.12	0.02	0.02	0.93
Tips	0.68	0.29	1.63	0.39
Afternoons	1.37	0.50	3.77	0.55
Trials	0.60	0.25	1.39	0.23
Fast	0.56	0.23	1.37	0.20
Master	0.67	0.34	1.33	0.26
Consultants	0.80	0.28	2.24	0.67
Easier	1.57	0.76	3.23	0.23
Obvious	1.79	0.73	4.39	0.20
Nurses	1.06	0.41	2.76	0.90
Hold	0.53	0.16	1.72	0.29
Journal	1.20	0.39	3.71	0.76
Rounder	0.70	0.27	1.77	0.45
Behavior	1.20	0.39	3.71	0.78
Procedure	0.85	0.27	2.69	0.79
Shadow	1.44	0.44	4.71	0.55
Wealth	0.68	0.33	1.39	0.29
Studies	0.83	0.35	1.94	0.66
Possibly	0.80	0.22	2.82	0.73
Stuff	0.80	0.28	2.23	0.67
Light	0.73	0.34	1.55	0.41
Keeps	0.50	0.22	1.15	0.10
Humble	1.50	0.59	3.79	0.40
Depth	0.63	0.29	1.35	0.23
Frequent	0.50	0.25	1.02	0.06
Didactic	0.77	0.33	1.78	0.54
Placed	0.55	0.21	1.45	0.22
Laid	0.80	0.42	1.50	0.48
Humor	0.61	0.40	0.94	0.03
Hearing	2.57	1.05	6.31	0.04

© 2019 Heath JK et al. *JAMA Network Open.*

Big	0.45	0.23		0.91
Constantly	0.44	0.22	0.87	0.03
Engaged	1.53	0.91	2.59	0.02
Pointed	2.69	0.83	8.76	0.11
Admissions	1.25	0.69	2.28	0.10
Committed	1.76	1.06	2.89	0.46
Talks	1.68	1,99	2.81	0.03
Data	0.88	0.50	1.52	0.05
Round	0.97	0.58	1.63	0.64
Ideal	0.96	0.45	2.04	0.91
Workload	0.87	0.35	2.16	0.91
Research	1.44	0.84	2.49	0.76
Fostered	1.42	0.73	2.77	0.18
Personality	0.32	0.14	0.74	0.30
Emphasis	0.48	0.24	0.93	0.008
Post	0.62	0.32	1.18	0.03
Someone	1.25	0.69	2.24	0.15
Demeanor	0.51	0.28	0.93	0.45
Lectures	1.50	0.97	2.33	0.03
Multiple	1.53	0.94	2.50	0.07
Otherwise	0.77	0.45	1.32	0.09
Privilege	1.47	0.88	2.44	0.35
Knowledgeable	1.34	1.06	1.70	0.14
Hands	1.27	0.64	2.52	0.01
Procedures	0.85	0.52	1.39	0.49
Seemed	1.05	0.64	1.73	0.51
Aspects	1.08	0.65	1.80	0.85
Real	0.88	0.56	1.41	0.77
Long	0.79	0.55	1.12	0.61
Based	0.60	0.44	0.82	0.20
Found	0.75	0.52	1.09	0.001
Evidence	0.70	0.51	0.96	0.13
Able	1.21	0.96	1.51	0.03
Incorporate	1.94	1.13	3.36	0.10
Members	1.12	0.82	1.54	0.02
Fellow	0.82	0.64	1.08	0.462
Knowledge	0.62	0.51	0.76	0.16
Teacher	1.10	0.98	1.23	0.01

© 2019 Heath JK et al. *JAMA Network Open.*

Get	1.41	1.13	1.76	1.76
Provided	1.10	0.84	1.43	0.002
Fellows	0.63	0.48	0.83	0.49
Patients	1.20	0.07	1.34	0.001
Time	1.07	0.95	1.19	0.001
Working	1.05	0.93	1.18	0.28
Service	1.12	0.87	1.20	0.41
Work	0.93	0.84	1.03	0.83
One	0.75	0.64	0.89	0.18
Pleasure	0.73	0.61	0.86	0.001
Plan	0.82	0.63	1.07	0.001
Spend	1.96	0.58	1.57	0.15
Amazing	1.37	1.06	1.78	0.86
Management	0.83	0.65	1,06	0.02
Wonderful	1.56	0.29	1.89	0.13
Plans	1.10	0.82	1.45	0.001
Hope	0.99	0.81	1.23	0.53
Interns	1.00	0.78	1.30	0.99
Make	1.10	0.90	1.32	0.99
Enthusiastic	1.74	0.25	2.44	0.36
Supportive	1.26	1.04	1.51	0.001
Approachable	1.09	0.88	1.35	0.02
Model	1.12	0.92	1.37	0.42
Fantastic	0.75	0.61	0.93	0.26
Comfortable	1.14	0.84	1.56	0.01
Busy	1.05	0.83	1.31	0.40
Friendly	0.36	0.24	0.52	0.71
Rapport	1.29	0.82	2.05	<0.001
Still	1.50	1.08	2.09	0.26
Career	1.16	0.73	1.89	0.02
Favorite	0.78	0.53	1.13	0.53
Nice	1.30	0.94	1.80	0.18
Allowed	0.90	0.71	1.15	0.11
Failure	1.01	0.61	1.68	0.41
Expectations	1.09	0.77	1.54	0.96
Person	1.18	0.80	1.72	0.64
Although	1.13	0.69	1.83	0.41
Wish	1.31	0.91	1.88	0.63

© 2019 Heath JK et al. *JAMA Network Open.*

Compassionate	0.92	0.60	1.41	0.15
Lead	1.34	0.92	1.97	0.71
Month	2.96	1.55	5.65	0.13
Preceptor	0.86	0.58	1.27	0.001
Inspiring	1.31	0.73	2.33	0.45
Question	0.62	0.36	1.10	0.37
Encouraged	1.88	1.31	2.69	0.10
Attitude	1.09	0.66	1.79	0.001
Follow	1.30	0.74	2.30	0.74
Want	1.00	0.59	1.69	0.36
Structured	1.30	0.59	2.84	0.99
Strong	0.69	0.44	1.09	0.52
Short	0.79	0.51	1.23	0.11
Detail	0.66	0.38	1.15	0.30
Mind	0.78	0.39	1.56	0.14
Glad	1.66	0.81	3.39	0.48
Benefit	0.60	0.28	1.26	0.17
Independent	1.07	0.63	1.82	0.18
Meet	0.94	0.43	2.07	0.80
Prepared	0.80	0.36	1.77	0.88
Seeing	1.11	0.65	3.36	0.58
Smoothly	0.82	0.40	1.66	0.01
Whenever	0.88	0.54	1.44	0.58
Pleasant	0.47	0.28	0.80	0.61
SAR	0.60	0.22	1.59	0.01
Oriented	0.72	0.35	0.27	0.30
Suggest	0.68	0.20	2.33	0.36
Believe	1.20	0.69	2.07	0.54
Positive	0.94	0.65	1.38	0.52
Proactive	0.65	0.24	1.76	0.76
Organized	1.52	0.91	2.55	0.40
Previous	1.05	0.38	2.89	0.11
Leading	0.96	0.49	1.85	0.93
Diagnoses	1.02	0.34	3.05	0.89
Remarkable	1.20	0.47	3.02	0.97
Initially	1.67	0.53	5.28	0.71
Styles	0.79	0.22	2.82	0.38
Engage	1.60	0.67	3.79	0.73

© 2019 Heath JK et al. *JAMA Network Open.*

Moving	1.33	0.54	0.54	0.29
Admired	2.63	0.91	7.59	3.27
Therapeutic	0.84	0.32	2.20	0.10
Emphasizes	0.75	0.24	2.28	0.72
Pressure	1.60	0.55	4.60	0.61
Documentation	0.27	0.06	1.23	0.39
Involve	0.12	0.2	0.93	0.10
Competent	0.60	0.20	1.74	0.04
Resources	1.20	0.42	3.41	0.35
Displayed	0.22	0.04	0.98	0.73
Warm	0.78	0.41	1.49	0.05
Via	0.40	0.11	1.47	0.45
Majority	0.40	0.11	1.47	0.17
Board	1.20	0.35	4.13	0.17
Versed	0.59	0.15	2.39	0.78
Walked	0.72	0.17	3.00	0.47
Worker	4.79	1.02	22.57	0.65
Wisdom	0.45	0.12	1.69	0.05
Delight	1.20	0.42	3.41	0.24
Empathetic	1.71	0.65	4.50	0.74
Text	0.60	0.25	1.40	0.28
Couple	0.72	0.26	1.97	0.23

© 2019 Heath JK et al. *JAMA Network Open.*
